# The workplace expectations and preferences of Gen Z nurses: exploratory factor analysis

**DOI:** 10.1186/s12912-025-03761-y

**Published:** 2025-08-27

**Authors:** Modi Al-Moteri, Emad Althobaiti, Ghaida M. Aljuaid, Mjd F. Alotaibe, Ruba A. Alharthi, Maryam A. Aljuaid, Shahad F. Alboqami, Jamil Aljuaid, Daniel Joseph E. Berdida, Rizal Angelo N. Grande, Manal Saleh Moustafa Saleh, Maaidah M. Algamdi, Mesheil Alalyani, Amani A. Alrehaili

**Affiliations:** 1 Medical Surgical Nursing Department, College of Nursing, Taif University, POB 11099, Taif, 21944 Saudi Arabia; 2 King Salman Specialized Hospital, Ministry of National Guard Health Affairs, Taif, 26573 Saudi Arabia; 3 Department of Case Management, Taif Health Cluster, Taif, 21944 Saudi Arabia; 4 Department of Nursing, North Private College of Nursing, Arar, Northern Border, Saudi Arabia; 5 Department of Nursing, Fakeeh College for Medical Sciences, Jeddah, Makkah, Saudi Arabia; 6 Nursing Administration, Faculty of Nursing, Zagazig University, Sharkia, Egypt; 7 Department of Nursing Sciences, College of Applied Medical Science, Shaqra University, Shaqra, Saudi Arabia; 8 Community and Psychiatric Nursing Department, Faculty of Nursing, University of Tabuk, Tabuk, Saudi Arabia; 9 Nursing College, King Khalid University, Khamis Mushait, Abha, Saudi Arabia; 10 Department of Clinical Laboratories Science, College of Applied Medical Science, Taif University, Taif, 21944 Saudi Arabia

**Keywords:** Generation Z nurses, Workforce, Sustainability, Policy reform

## Abstract

**Background:**

Gen Z will comprise a significant portion of the nursing workforce over the next decade and beyond. The workplace expectations of Gen Z nurses may not align with the realities of nursing environments, which can ultimately impact their ability to engage and thrive in their roles. Therefore, it is essential to understand this generation to help healthcare systems better prepare to engage and retain them. This study aims to explore the workplace expectations and preferences of Gen Z nurses born between 1997 and 2005.

**Methods:**

A cross-sectional design was employed. A survey was developed to identify the core aspects of Gen Z nurses’ workplace expectations. Due to the exploratory nature of the study and the absence of predefined hypotheses, it was essential to assess the factorial validity of the adapted instrument. Therefore, exploratory factor analysis (EFA) was conducted to uncover the latent structure of the 45-item scale, providing data-driven insights into how Gen Z nurses conceptualize workplace expectations. The items were drawn from two validated sources: (1) the Work Value and Attitude Scale and (2) the Healthcare Career Choice Scale.

**Results:**

EFA revealed six key factors that influence professional priorities: (1) Supportive, stable, and growth-oriented workplace, (2) Collaborative practice and teamwork, (3) Bureaucratic resistance and tech critique, (4) Age-based disrespect in the workplace, and (5) Logistical convenience and job perks, and (6) Professional identity and career growth. Together, these factors accounted for 62.21% of the total variance, demonstrating excellent internal consistency with a Cronbach’s alpha of 0.83 for the entire instrument, while subscale alphas ranged from 0.91to 0.72. The results reflect the multifaceted nature of nurses’ workplace experiences, including environmental support, interprofessional collaboration, career aspirations, resistance to structural barriers, and generational challenges.

**Conclusions:**

This research has the potential to significantly inform policymakers on how to improve job satisfaction and professional development for Gen Z nurses.

**Clinical trial number:**

Not applicable.

## Introduction

Generation Z (Gen Z), those born after 1995 [1], is recognized for its entrepreneurial mindset, inclusivity, and strong preference for meaningful work [2]. They stand apart from earlier generations, particularly in their work interactions and expectations [3]. This generation is driven to pursue ideal jobs and seek out skill-enhancing opportunities, indicating that they will change jobs more frequently than previous generations. If they are dissatisfied with something, they are prepared to make an immediate change [4]. This creates a challenge not only in attracting Gen Z but also in focusing efforts on meeting their needs to promote retention.

In recent decades, the global nursing workforce has experienced significant evolution and been profoundly impacted by several events, including the COVID-19 pandemic [5]. The nursing workforce possesses many strengths, but it is beginning to face substantial challenges that are already impacting nurses and the healthcare systems and organizations in which they operate. These challenges stem from societal changes that lead to increased demand for healthcare, dynamics within the nursing and broader healthcare workforce, and health-related public policies, along with various factors influencing the quantity, distribution, diversity, and educational readiness of nurses [6].

In Saudi Arabia, although the nursing workforce continues to grow and evolve, it is facing significant challenges that may impact workforce stability and healthcare delivery. These challenges include the rising demand for healthcare services driven by demographic shifts, increased prevalence of chronic diseases, and national healthcare reforms under Vision 2030 [7]. Additionally, workforce dynamics, such as a high reliance on expatriate nurses and efforts toward Saudization, create new complexities related to workforce distribution, retention, professional development, and leadership preparedness [8].

Numerous studies conducted globally have examined the attitudes of Gen Z toward work across various sectors, including nursing [9–11]. Some studies emphasize intragenerational differences, indicating that the visions, preferences, and traits of Gen Z can vary by region [12] and may also be shaped by their views on the workplace [13]. Most existing studies have been conducted in Western contexts [14], with limited data examining how Gen Z nurses’ professional priorities might manifest in non-Western healthcare systems like those in Saudi Arabia. Given the rapid entry of Gen Z nurses into the Saudi workforce, there is a continued need to empirically investigate the specific factors shaping their workplace expectations to guide effective workforce planning, management, and retention strategies. Therefore, it is essential to understand this generation to help the healthcare system better prepare to engage and retain them. This study aims to explore the workplace expectations and preferences of Gen Z nurses born between 1997 and 2005. The following research questions were examined:

1. What are the underlying dimensions of workplace expectations for Gen Z nurses in Saudi Arabia?
2. Do the identified factors exhibit adequate internal consistency and reliability?

## Methods

This study employed a cross-sectional design. Given the study’s purpose of exploring the workplace expectations and preferences of Gen Z nurses, current tools evaluating Gen Z’s work values are often fragmented, specific to certain contexts, or designed for populations outside of healthcare. Kanste et al. [14] highlighted that healthcare professionals from Gen Z possess intricate and distinct expectations that are not adequately met by existing instruments. To develop a holistic understanding of the workplace expectations and preferences of Gen Z nurses within the specific cultural and professional context of nursing in Saudi Arabia, a new instrument was created to ensure both content validity and contextual relevance for this study group.

### Participants

Participants included nurses in their first and second years of clinical employment, as well as prospective nurses in their third, fourth, and internship study years. Generation Z was operationally defined as individuals born between 1997 and 2005, encompassing those aged 18 to 27 as of August 2023. This age range was selected to include younger members of Generation Z who are either entering the workforce or in advanced stages of their nursing education. The exclusion of individuals under 18 ensured that all participants could provide informed consent independently, as individuals below this age are considered minors in Saudi Arabia and would require parental permission to participate.

### Survey development and pretesting

The development and validation of the survey instrument included several steps to ensure it comprehensively addressed the study’s aim while maintaining clarity, reliability, and relevance. The steps are outlined below.

#### Step 1: literature search

A thorough literature search was conducted u*s*ing databases such as PubMed and Web of Science. The search terms encompassed combinations of “Generation Z,” “nursing,” “work values,” “career attitudes,” “career choices,” “career expectation,” and “validated scales”. Consequently, two validated instruments suitable for adaptation were identified: the Work Value and Attitude Scale [10] and the Healthcare Career Choice Scale [15]. The integration of these two scales was guided by Generational Cohort Theory, which posits that individuals within the same generational cohort develop shared work-related attitudes, values, and career motivations shaped by common sociohistorical experiences [16, 17]. As prior research indicates, Gen Z exhibits distinct preferences across multiple domains, including work values, leadership expectations, job motivators, and career choices [18, 19]. Merging these instruments provided a comprehensive framework to capture both intrinsic work values and extrinsic career motivations relevant to Generation Z nurses, aligning with the study’s focus to holistically assess their professional expectations.

The first tool, developed by Tan and Chin [10], examines generational differences in nurses’ work values, engagement, and job satisfaction. This instrument was chosen for its focus on understanding the attitudes and behaviors of Generation Z, a key aspect for this research. The second tool, created by Almutary and Al-Moteri [15], explores factors influencing career choices in healthcare. It was selected for its comprehensive approach to identifying motivations and aspirations in career decision-making, which is central to understanding the career choices of Generation Z nurses. Since the original instruments were administered in their validated English versions, no translation or back-translation process was required.

#### Step 2: expert review of items

To establish Item-level Content Validity Index (I-CVI), the tool was first sent to five expert reviewers, all of whom are academic researchers. Two reviewers specialize in cognitive psychology, two nursing educators, and one is an expert in instrument development. The five reviewers independently received 68 items drawn from the two selected scales. They were asked to rate items that align with the research aim and could contribute to understanding the career motivations, work preferences, and expectations of Gen Z nurses on a 4-point relevance scale: 1 (not relevant), 2 (somewhat relevant), 3 (quite relevant), and 4 (highly relevant).

To quantify the content validity, the level of agreement among the five experts regarding the relevance of a specific item was calculated based on the proportion of experts rating the item as either 3 (quite relevant) or 4 (highly relevant). A total of 41 items (60.3%) demonstrated full agreement (I-CVI = 1.00), while four items (5.9%) showed partial agreement (I-CVI = 0.80) and were included. Meanwhile, the remaining 23 items (33.8%) demonstrated lower agreement (I-CVI ≤ 0.60) and were excluded. The Scale-Level Content Validity Index, calculated as the average of all I-CVIs (S-CVI/Ave), was 0.729, indicating adequate content validity and supporting the suitability of the instrument for subsequent psychometric evaluation [20].

The final version of the instrument consisted of 45 items, carefully distributed across the five research themes:

1. What type of work environment does Generation Z favor?: This section comprised 16 items addressing key factors such as workplace flexibility, leadership quality, work-life balance, and collegial relationships.
2. What are their expectations for an ideal nursing career?: Twelve items explored areas like job satisfaction, financial security, career growth opportunities, and intrinsic rewards.
3. How do they envision their professional trajectories?: Nine items focused on aspirations, long-term goals, and their vision for professional identity.
4. What are their perspectives on empowerment and leadership in nursing?: Five items examined their views on decision-making authority, collaboration, and leadership engagement.
5. How do they perceive the role of technology in nursing?: Three items captured their attitudes toward the integration of technology in nursing practice, including its benefits and challenges.

#### Step 3: pilot testing

Following the I-CVI, the survey instrument underwent pilot testing with a sample of 30 nursing interns. The primary aim of the pilot study was to evaluate the clarity, usability, and reliability of the survey in a real-world setting. This phase was crucial for ensuring that the instrument could effectively capture the required data without introducing confusion or contributing to respondent fatigue. The survey was designed with a 5-point Likert scale for all items, ranging from 1 (“Not important at all”) to 5 (“Extremely important”).

On average, participants completed the survey within 10 min, confirming its brevity, user-friendly design, and logical structure. Feedback from participants further indicated that all items were clear, comprehensible, and relevant to the study aim. Consequently, no further modifications to the instrument were required following the pilot testing phase. To assess the instrument’s reliability, internal consistency was evaluated using Cronbach’s alpha. The resulting value of 0.95 indicated excellent reliability [21], demonstrating that the survey items effectively measured the intended constructs. This high-reliability score reinforced the instrument’s readiness for broader application in the main study, ensuring its capability to generate meaningful and consistent data.

### Data collection

For an interpretable factor structure to emerge, the literature suggests using at least 5 participants per survey item [22, 23]. Given that there are 45 items on the current survey, a minimum of 225 participants is required for exploratory factor analysis.

Participants were recruited through convenience sampling via an online Google Forms survey. The survey link was distributed through social media platforms and ran from November 1, 2024, to November 25, 2024, concluding upon reaching the target sample size. Participants provided electronic consent for their participation in this research. Data was also collected in-person, as one of the researchers visited various healthcare settings to invite eligible participants to join the study. Confidentiality and anonymity were maintained . Additionally, participation in this study was voluntary, and participants could withdraw at any time. The data collection period lasted four weeks, yielding 262 eligible responses.

### Ethical approval

This study was reviewed and approved by the Scientific Research Ethics Committee in King Faisal Medical Complex (KFMC) (IRB number: 2024-E-92). Online informed consent was obtained from all the participants.

### Data analysis

Given the exploratory nature of this study, which aimed to uncover the underlying structure of workplace expectations and preferences among Gen Z nurses, the combination of descriptive statistics and exploratory factor analysis (EFA) was appropriate and sufficient. According to Fabrigar and Wegener [24], EFA is suitable when the study aim is to uncover the dimensional structure without predefined hypotheses. EFA provided data-driven insights into the dimensional structure of the measured variables, while descriptive statistics offered detailed profiles of participant responses. Since the study’s aim was not to test specific hypotheses or examine group differences, inferential statistical analysis was not required at this stage.

Data were analyzed using SPSS version 16.0 (Statistical Package for the Social Sciences). Descriptive statistics, including frequencies and percentages, were calculated to summarize demographic characteristics and what Gen Z nurses expect and prefer. Principal component analysis with varimax rotation and Kaiser normalization was used, retaining factors with eigenvalues greater than 1 and item loadings of 0.50 or higher. Factors were considered significant if they explained at least 4% of the total variance. To determine whether the 45 items represented an internally consistent measure, the researcher computed an overall Cronbach alpha (α) coefficient.

### Results

A total of 262 surveys were initially collected during the data collection period. After thorough review, 19 surveys were excluded due to incomplete responses or missing critical data, resulting in a final dataset of 243 valid surveys for analysis. This final dataset met the minimum sample size requirements for robust statistical analysis and ensured that only complete and reliable responses were included in the study.

### Sociodemographic characteristics

As shown in Table 1, the sample was predominantly young, with participants born between 2000 and 2002 constituting the largest group (50.6%), followed by those born between 2003 and 2005 (41.2%), and a smaller proportion born between 1997 and 1999 (8.2%). Female respondents comprised the majority of the sample (63.8%). In terms of professional roles, the largest groups included nursing interns (25.1%), fourth-year nursing students (24.3%), and third-year nursing students (21.8%). A smaller number of participants were employed as staff nurses, with 21.0% having one year of experience, 4.1% with three years, and 3.7% with two years.

Only 30.5% of participants believe their nursing careers ‘very much’ meet their expectations, which is less than one-third. Meanwhile, the largest group (58.4%) responded ‘Somewhat,’ indicating that their nursing careers meet some expectations but may fall short in key areas. The data on career advancement goals show that most respondents are eager for professional growth. Specifically, 25.1% aim to pursue specialized roles like Nurse Practitioner, while 20.6% seek leadership positions such as Nurse Manager. Additionally, 18.1% are interested in transitioning to education or research, and 12.3% plan to continue their nursing education, indicating strong ambitions for academic and knowledge-based progress. A small portion, 5.3%, intends to remain at their current level, and 5.3% plan to leave clinical care. Meanwhile, 14% are uncertain about their future. Overall, the findings suggest a workforce motivated to develop within or near the nursing field.

**Table 1:** Sociodemographic variables (*n* = 243)

Variable		*n*	%
Date of birth	1997 to 1999	20	8.2%
	2000 to 2002	123	50.6%
	2003 to 2005	100	41.2%
Gender	Female	155	63.8%
	Male	88	36.2%
Designation	1 year of employment as a Staff Nurse	51	21.0%
	2 years of employment as a Staff Nurse	9	3.7%
	3 years of employment as a Staff Nurse	10	4.1%
	3rd Year Nursing Student	53	21.8%
	4th Year Nursing Student	59	24.3%
	Nursing Intern	61	25.1%
Current job meets expectations	Not at all	27	11.1%
	Somewhat	142	58.4%
	Very much	74	30.5%
Career advancement goals	Pursuing a specialized role (e.g., Nurse Practitioner)	61	25.1%
	Pursuing further education in nursing	30	12.3%
	Pursuing leadership roles (e.g., Nurse Manager)	50	20.6%
	Shifting to education/research	44	18.1%
	Staying at current level	13	5.3%
	Transitioning out of clinical care	11	4.5%
	Unsure	34	14.0%

### Factor analysis

To address the first research question concerning identifying the underlying dimensions of workplace expectations for nurses in Saudi Arabia, EFA was conducted and presented below. The Kaiser-Meyer-Olkin (KMO) test yielded a value of 0.953, indicating excellent sampling adequacy as per Kaiser’s (1974) criteria, and Bartlett’s Test of Sphericity was significant (χ² = 6545.798, df = 703, *p* < .001), confirming the dataset’s suitability for factor analysis (Table 2).

**Table 2:** KMO and bartlett’s test

Kaiser-Meyer-Olkin Measure of Sampling Adequacy.		0.953
Bartlett’s Test of Sphericity	Approx. Chi-Square	6545.798
	df	703
	Sig.	< 0.001

Using Maximum Likelihood extraction and Varimax rotation yielded six components, which together explained 62.21% of the total variance, indicating a strong and multifactorial structure underlying the data. As detailed in Table 3, the first factor, supportive, stable, and growth-oriented workplace, accounted for 20.8% of the variance, followed by collaborative practice and teamwork (12.9%), professional identity and career growth (10.6%), bureaucratic resistance and tech critique (8.1%), logistical convenience and job perks (4.9%), and age-based disrespect in the workplace (4.6%). These findings demonstrate a robust multidimensional structure underlying Generation Z nurses’ workplace expectation.

**Table 3:** Total variance explained

Component	Initial Eigenvalues			Rotation Sums of Squared Loadings		
	Total	% of Variance	Cumulative %	Total	% of Variance	Cumulative %
1. Supportive, stable, and growth-oriented workplace	19.011	42.246	42.246	9.379	20.842	20.842
2. Collaborative practice and teamwork	3.366	7.481	49.726	5.821	12.935	33.776
3. Professional identity and career growth	2.031	4.514	54.241	4.799	10.665	44.441
4. Bureaucratic resistance and tech critique	1.259	2.798	57.039	3.685	8.190	52.631
5. Logistical convenience and job perks	1.210	2.689	59.727	2.222	4.939	57.569
6. Age-based disrespect in the workplace	1.117	2.483	62.210	2.088	4.640	62.210

The rotated component matrix, presented in Table 4, demonstrates the specific variables that load significantly onto each component, offering detailed insights into the core themes. Among the 45 expectation variables, 37 were retained in six components.

**Table 4:** Rotated factor Matrix^a^

	Component					
	1	2	3	4	5	6
2. Working in a supportive, relaxed, and positive nursing environment.	0.771					
8. Avoiding frequent overtime and long shifts is crucial for maintaining my well-being and work-life balance.	0.771					
9. Having flexibility in my work schedule, such as choosing start and end times, enhances my job satisfaction.	0.749					
7. Maintaining a positive professional image among colleagues, patients, and supervisors is essential for my career growth.	0.694					
12. Clear direction within my nursing unit or team enhances my work performance.	0.678					
10. Feeling comfortable expressing my opinions and suggestions within the care environment is essential for fostering collaboration.	0.664					
5.The comfort and layout of the healthcare facility directly impact my work experience	0.639					
22. Ensuring job stability and security within the nursing profession.	0.624					
17. Continuously advancing my nursing knowledge and skills.	0.622					
3. Maintaining manageable stress levels in my nursing responsibilities.	0.617					
45. I find that using the latest technology improves my ability to provide better patient care.	0.589					
1. Engaging in diverse nursing tasks that offer variety and reduce boredom.	0.581					
42. I believe that effective collaboration among healthcare professionals enhances patient outcomes.	0.562					
23. Working for a healthcare institution with a strong reputation and solid foundation.	0.548					
19. Starting my nursing career with a competitive salary that reflects my qualifications.	0.507					
41. My contributions to team discussions are respected and considered in decision-making.		0.780				
40. I am regularly involved in interprofessional meetings to discuss patient care strategies.		0.762				
38. I actively participate in collaborative decision-making processes within my healthcare team.		0.755				
39. Our nursing team works together to develop and implement patient care plans.		0.658				
37. I feel comfortable advocating for my patients’ needs within the healthcare team.		0.655				
36. I am encouraged to take the initiative in addressing patient care challenges.		0.554				
34. My opinions and suggestions are valued by my colleagues and supervisors in clinical settings.		0.522				
20. Having access to a mentor who supports my professional and personal development.			0.569			
11. I perform best when there is strong leadership in place.			0.533			
15. Finding fulfillment and purpose in my work as a nurse.			0.532			
18. Achieving potential for competitive earnings and financial growth in my nursing career.			0.525			
27. I aspire to take on leadership roles (e.g., charge nurse, nurse manager) in the short to medium term.			0.505			
31. I believe some rules in healthcare are made to be broken.				− 0.751		
32. I have a low tolerance for unnecessary bureaucracy and ineffective policies in hospitals.				− 0.721		
30. I tend to challenge traditional nursing norms, such as uniforms, scheduling, and relationships with supervisors.				− 0.660		
44 I worry that technology could replace certain aspects of nursing care.				− 0.590		
43 Technology, like health electronic records (HER) systems and medical devices, makes my nursing tasks harder.				− 0.516		
35. I have the authority to implement changes in patient care based on my professional judgment.				0.508		
4. Living close to my workplace is essential for maintaining a healthy work-life balance					0.591	
6. Receiving practical benefits like scrubs and professional tools adds value to my job.					0.515	
14. Younger nurses are often not respected because of their perspective.						− 0.813
13. Younger nurses are often treated dismissively by senior staff.						− 0.768

Extraction Method: Principal Component Analysis

Rotation Method: Varimax with Kaiser Normalization

a. Rotation converged in 10 iterations

### Internal consistency and reliability

To address the second research question and evaluate the reliability of the identified factors, internal consistency was assessed using Cronbach’s alpha coefficients for each subscale. As shown in Table 5, all six factors demonstrated acceptable to excellent internal consistency, ranging from 0.91 to 0.72, with a Cronbach’s alpha of 0.83 for the entire instrument. These results indicate that the survey subscales are internally consistent and reliably measure the intended outcome constructs.

**Table 5:** Internal consistency and reliability

No.	Interpreted Label	Items (Assigned by Highest Loading)	Adjusted Cronbach’s Alpha
1	Supportive, stable, and growth-oriented workplace	15	0.891
2	Collaborative practice and teamwork	7	0.895
3	Professional identity and career growth	5	0.81
4	Bureaucratic resistance and tech critique	6	0.91
5	Logistical convenience and job perks	2	0.74
6	Age-based disrespect in the workplace	2	0.72

## Discussion

This study aimed to explore the underlying factors shaping workplace expectations and preferences among Gen Z nurses. Rather than focusing on group comparisons or inferential statistical testing, the primary focus was to provide a comprehensive understanding of the overall structure and key dimensions that characterize Gen Z nurses’ professional priorities. The study employed a structured approach, utilizing validated instruments, expert review, and exploratory factor analysis to uncover the multidimensional structure of these expectations. The factor analysis identified six main dimensions that shape Gen Z nurses’ workplace expectations and preferences: (1) Supportive, stable, and growth-oriented workplace, (2) Collaborative practice and teamwork, (3) Bureaucratic resistance and tech critique, (4) Age-based disrespect in the workplace, and (5) Logistical convenience and job perks, and (6) Professional identity and career growth.

These dimensions reflect the diverse priorities of Gen Z nurses, combining their desire for personal and professional growth with a strong preference for collaboration and transparent communication. This focus aligns with existing generational literature highlighting Gen Z’s emphasis on career development, teamwork, and maintaining a healthy work-life balance [9, 25, 26].

Gen Z nurses seem to prefer ongoing development opportunities [27], flexible schedules [28], and clear career advancement paths [9], emphasizing their long-term professional goals. These findings are consistent with Abujaber et al. [29], reinforcing the importance of workplace stability and clarity in meeting this generation’s expectations.

Notably, Gen Z nurses tend to question and challenge institutional norms. They show low tolerance for bureaucratic inefficiencies and ineffective policies [14]. Their belief that some healthcare rules should be broken, combined with a willingness to challenge traditional nursing practices—such as dress codes, scheduling, and supervisory structures—reflects a desire for greater flexibility and autonomy. These insights are crucial for healthcare leaders seeking to cultivate organizational environments that align with the distinct values and expectations of this emerging nursing workforce.

Interestingly, while earlier research [10] indicates that Gen Z nurses are generally at ease with technology and often act as advocates for it within healthcare systems, the current study shows a more hesitant viewpoint among Gen Z nurses in this sample. Even though they value innovation, there are ongoing concerns about technology potentially replacing elements of patient care and leading to bureaucratic complexities. These discrepancies might be influenced by context or regional factors specific to the healthcare landscape in Saudi Arabia [12], including variations in technology integration, organizational culture, or available training opportunities. Therefore, these findings underscore the importance of considering cultural and organizational contexts when evaluating generational attitudes toward technology in healthcare.

The findings of the current study highlight that Gen Z nurses feel their opinions are frequently undervalued in clinical settings by other nurses of different generations, suggesting a generational gap that might hinder teamwork, mentorship, and knowledge exchange within healthcare teams. This issue is well documented in the literature; for example, Tan and Chin [10] found that younger nurses often face a lack of respect from older colleagues, which contributes to conflict and lower engagement in healthcare teams. Furthermore, Alzuman and Alzouman [30] reported that variations in perceived control, responsibility, and interaction opportunities highlight broader generational differences. Alzuman and Alzouman underline the importance for nurse managers and hospital leaders to implement more inclusive approaches that consider generational diversity in leadership, communication, and decision-making.

It may be crucial for leadership to address key areas such as maintaining a stable, growth-focused workplace, promoting collaborative teamwork, and respecting younger professionals. Additionally, reducing bureaucratic inefficiencies, limiting overreliance on technology, and enhancing logistical support and job benefits can better align organizational practices with generational expectations. Investing in professional identity development and providing long-term career advancement opportunities can significantly boost job satisfaction, engagement, and retention. By concentrating on these six key factors, organizations will not only adapt to an evolving workforce but also improve patient care and organizational resilience [4, 31].

In this study, internal consistency reliability was used to evaluate how well the items within each identified factor measured the underlying constructs of Gen Z nurses’ workplace expectations. The subscales demonstrated good to excellent internal consistency, as indicated by their Cronbach’s alpha values. These findings affirm the reliability of the derived factor structure and suggest that the items within each subscale are coherent and effectively capture distinct yet related dimensions of professional priorities among Gen Z nurses [21].

### Implications

The findings of this study offer valuable implications for nursing practice, leadership, and workforce development within Saudi Arabia and globally. In the context of Saudi Arabia, as the healthcare sector evolves, aligning workplace practices with Gen Z nurses’ expectations is essential for fostering a resilient and locally empowered workforce. This includes fostering supportive work environments, promoting collaboration, offering professional growth, and ensuring regular recognition and feedback. Worldwide, as healthcare systems encounter similar generational shifts, these insights emphasize the need to adapt workforce strategies to meet the changing expectations of younger nurses. Addressing issues such as professional development, bureaucracy, work-life balance, and technology integration is vital for building a resilient, motivated, and future-proof nursing workforce across diverse healthcare settings.

### Limitations

Like any research, this study has its limitations. While efforts were made to include a diverse group of Gen Z nurses, the sample mainly consisted of nursing students and early-career nurses, primarily those in their third or fourth year of study. This focus may restrict the applicability of the findings to fully employed Gen Z nurses with more significant professional experience. Additionally, data were gathered via a self-administered online survey using convenience sampling, which could introduce selection bias and impact the overall representativeness of the sample. Therefore, caution is advised when applying these results to the wider population of Gen Z nurses across different practice environments and stages of their career development.

While the sample size was sufficient for factor analysis, the results may not completely represent the views of all Gen Z nurses across various healthcare settings in Saudi Arabia. Additionally, the study took place within a particular regional and organizational context, which might restrict its generalizability to other healthcare systems, such as private sector facilities or different cultural settings. Future research should aim for larger, more diverse samples across various healthcare organizations and regions to improve generalizability. Moreover, while this study aimed to identify the essential dimensions of workplace expectations, future investigations could examine how socio-professional demographic variables influence these expectations, thereby enhancing the understanding of potential differences among subgroups.

## Conclusion

This study identified key dimensions underlying workplace expectations among Generation Z nurses, including work environment, collaboration, recognition, professional relationships, and leadership opportunities. The findings provide insights into the priorities of this emerging workforce and highlight areas where healthcare organizations may consider targeted strategies to support job satisfaction, retention, and professional development. While the results contribute to understanding Gen Z nurses’ workplace perspectives, further research across broader contexts is warranted to validate and expand these findings.

## Data Availability

The author confirms that all data generated or analysed during this study are included in this manuscript.

## Abbreviations

Gen Z: Generation Zers
HCC Scale: Healthcare Career Choice Scale
KMO: Kaiser-Meyer-Olkin
EHRs: Electronic health records
AR: Augmented reality
AI: Artificial intelligence
